# *Cuscuta chinensis* and *C. campestris* Attenuate Scopolamine-Induced Memory Deficit and Oxidative Damage in Mice

**DOI:** 10.3390/molecules23123060

**Published:** 2018-11-22

**Authors:** Ming-Kuem Lin, Meng-Shiou Lee, Hui-Chi Huang, Tun-Jen Cheng, Yih-Dih Cheng, Chi-Rei Wu

**Affiliations:** 1 Department of Chinese Pharmaceutical Sciences and Chinese Medicine Resources, China Medical University, Taichung 40402, Taiwan; linmk@mail.cmu.edu.tw (M.-K.L.); leemengshiou@mail.cmu.edu.tw (M.-S.L.); hchuang@mail.cmu.edu.tw (H.-C.H.); p0912789107@gmail.com (T.-J.C.); 2 Department of Pharmacy, China Medical University Hospital, Taichung 40402, Taiwan; M99553@mail.cmuh.org.tw

**Keywords:** Cuscutae Semen, *Cuscuta campestris*, *Cuscuta chinensis*, memory-improving properties, scopolamine

## Abstract

The seeds of *Cuscuta chinensis* Lam. and *C. campestris* Yuncker have been commonly used as Chinese medical material for preventing aging. Our previous studies have found that *C. chinensis* and *C. campestris* possess anti-inflammatory activities in rodents. However, their other biological activities, such as memory-improving properties, have not yet been explored. In the present study, we examined the memory-improving effects of the extracts of *C. chinensis* and *C. campestris* on scopolamine (SCOP)-induced memory deficit and explored their underlying mechanism in mice. Both *Cuscuta* species improved SCOP-induced memory deficits in the passive avoidance test, elevated plus-maze, and spatial performance test of the Morris water maze in mice. In addition, compared with mice injected with SCOP, mice pretreated with both *Cuscuta* species stayed for a longer time on the platform for the probe test of the Morris water maze. Moreover, both *Cuscuta* species reduced brain acetylcholinesterase activity and malondialdehyde levels that were increased by SCOP, and the species restored the activities of antioxidant enzymes (superoxide dismutase and catalase) and the levels of glutathione that were decreased by SCOP in the brains of mice. Both *Cuscuta* species further decreased brain interleukin-1β and tumor necrosis factor-α levels that were elevated by SCOP. We demonstrated that both *Cuscuta* species exhibited a protective activity against SCOP-induced memory deficit, cholinergic dysfunction, oxidative damage, and neuroinflammation in mice, and *C. campestris* has better potential than *C. chinensis*. In addition, we provided evidence that the seeds of *C. campestris* can be used as Cuscutae Semen in Traditional Chinese Medicine.

## 1. Introduction

Alzheimer’s disease (AD), a progressive neurodegenerative disorder, endures major clinical symptoms such as behavioral and cognitive decline. These symptoms are caused by three major and neuropathological mechanism including the degeneration of cholinergic neuronal circuits in the basal forebrain (BF), extracellular amyloid β peptide (Aβ) deposition, and intracellular neurofibrillary tangles formation from hyperphosphorylated tau protein [1]. Recent studies have speculated that oxidative stress and/or inflammatory processes are also important biochemical factors in the above neuropathological characterization [2]. Accumulation of free radical damage and alterations in the activities of brain antioxidant enzymes such as superoxide dismutase (SOD) and catalase have been observed in AD patients [3]. When oxidative damage to lipids and proteins increased, the severity of cholinergic neuronal loss and deficits in memory and behavior also increased [4,5]. Learning and memory processes usually involve the activation of neurotransmitters such as acetylcholine and monoamines [6]. According to Myhrer’s meta-analysis of studies on four behavioral tasks [7], the acetylcholinergic system has a high influence on learning and memory. Scopolamine (SCOP), a muscarinic receptor antagonist, impairs learning and memory in rodents and humans mainly by decreasing cholinergic activity [8,9]. Some recent reports have indicated that SCOP causes cerebral oxidative damage and neuroinflammation, which are the same as the neuropathological mechanism of AD [10,11]. Hence, SCOP has been used to induce amnesia and/or dementia in experimental rodent models.

*Cuscuta chinensis* Lam. and *C. campestris* Yuncker belong to the genus *Cuscuta* of the Cuscutaceae family. The seeds of *C. chinensis* (Cuscutae Semen), which is recorded in the famous book *Shen Nong’s Herbal* and other Chinese traditional pharmacopoeias, have been commonly used as medicine to nourish the kidney and liver in Traditional Chinese Medicine. However, *C. campestris* Yuncker, which is widely distributed in the wild, is not recorded in pharmacopoeias in Taiwan. Many studies have shown that *Cuscuta* species have various biological activities, including antibacterial, antimicrobial, antioxidant, anti-inflammatory, antiproliferative, hepatoprotective, and antiulcer activities [12,13,14]. *C. chinensis* reduced the levels of nitric oxide and malondialdehyde (MDA) by increasing the activities of antioxidant enzymes in the liver [13], and it reduced the levels of inflammatory mediators and proinflammatory cytokines in lipopolysaccharide-stimulated BV-2 microglia and RAW264.7 cells [15,16]. *C. chinensis* also improved the learning-memory ability in AD mice with hypoendocrinism [17]. Flavonoids of *C. chinensis* exerted the protective effects on cerebral ischemia–reperfusion injury by inhibiting inflammation in brain tissue [18]. *C. campestris* exhibited antioxidant activity in vitro as well as antifungal activity [19], and it reduced the MDA level by increasing the activities of antioxidant enzymes in the liver [20]. These findings reveal that both *C. chinensis* and *C. campestris* possess antioxidant and anti-inflammatory activities. Previous studies have shown that quercetin 3-*O*-β-d-apiofuranosyl-(1-2)-β-d-galactoside, hyperoside, quercetin, and kaempferol are the major compounds in the seeds of *C. chinensis*, and the same compounds, excluding kaempferol, are present in the seeds of *C. campestris*. However, the contents of these compounds are different in the seeds of the two species [13,16,21]. This study evaluated whether *C. campestris* can be a substitute for *C. chinensis* for preventing aging. Due to the pathological mechanisms of AD, including oxidative stress and neuroinflammation, *C. chinensis* and *C. campestris* might possess brain neuroprotective effects. Therefore, this study examined and compared their efficacies and explored their mechanisms for their antioxidant and anti-inflammatory potential in SCOP-induced memory deficits in mice.

## 2. Results

### 2.1. Effects of C. chinensis and C. campestris Extracts on Locomotor and Exploratory Activities in SCOP-Treated Mice

**Figure 1:**
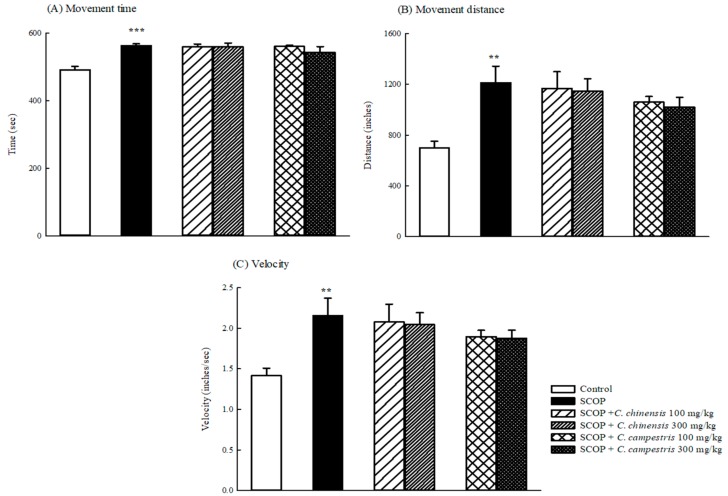
The effects of *Cuscuta chinensis* and *C. campestris* extracts (100 and 300 mg/kg, po) on (**A**) movement time, (**B**) movement distance, and (**C**) velocity in scopolamine (SCOP, 1 mg/kg, ip)-treated mice. Columns indicate mean ± SEM (*n* = 7). ** *p* < 0.01, *** *p* < 0.001 compared with the control group.

**Figure 2:**
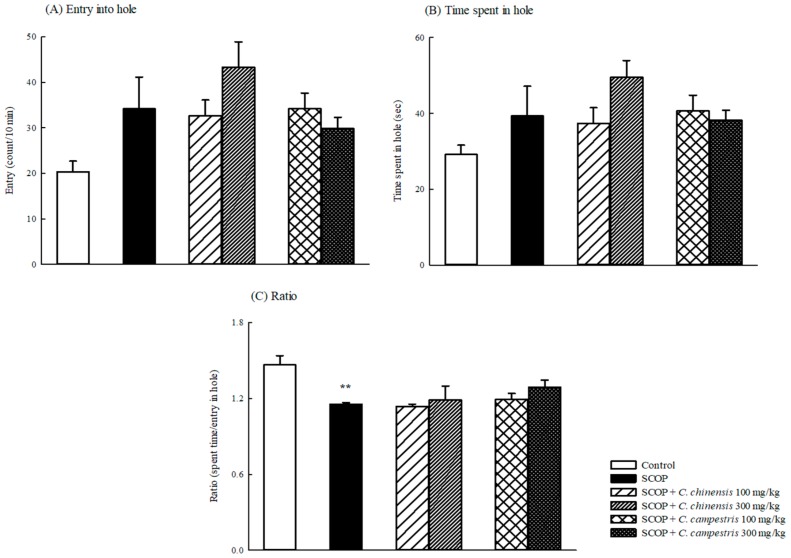
The effects of *Cuscuta chinensis* and *C. campestris* extracts (100 and 300 mg/kg, po) on (**A**) entries into hole, (**B**) time spent in hole, and (**C**) ratio of the time spent to the entry number in scopolamine (SCOP, 1 mg/kg, ip)-treated mice. Columns indicate mean ± SEM (*n* = 7). ** *p* < 0.01 compared with the control group.

Compared with the control group, SCOP increased the movement time and distance and then accelerated the movement velocity (Figure 1). The *C. chinensis* or *C. campestris* extract at any dose did not alter the higher locomotor activity caused by SCOP (Figure 1). SCOP slightly increased the number of entries and the time spent in the hole, but no significant difference was observed between the SCOP and control groups. However, SCOP decreased the ratio of the time spent to the entry number in the hole test in mice (Figure 2). The *C. chinensis* or *C. campestris* extract did not change the behavioral alteration caused by SCOP (Figure 2).

### 2.2. Effects of C. chinensis and C. campestris Extracts on Memory Deficits in SCOP-Treated Mice

**Figure 3:**
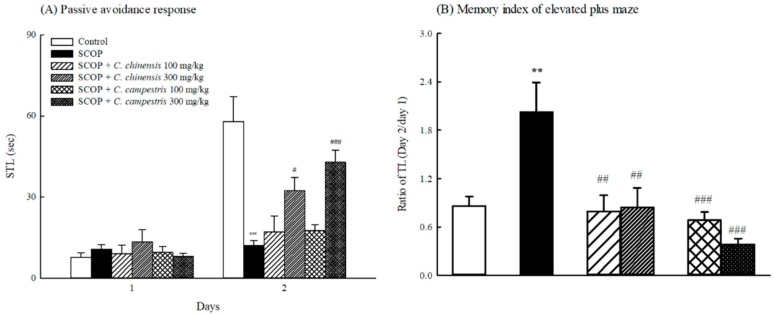
The effects of *Cuscuta chinensis* and *C. campestris* extracts (100 and 300 mg/kg, po) on (**A**) passive avoidance response and (**B**) memory index on elevated plus-maze in scopolamine (SCOP, 1 mg/kg, ip)-treated mice. Columns indicate mean ± SEM (*n* = 7). ** *p* < 0.01, *** *p* < 0.001 compared with the control group. # *p* < 0.05, *## p* < 0.01, ### *p* < 0.001 compared with the SCOP group.

Compared with the control group, SCOP shortened the step-through latency (STL) of the retention trial (Day 2 of the passive avoidance test) (Figure 3A). The *C. chinensis* or *C. campestris* extract at 300 mg/kg prolonged the STL of the retention trial that was shortened by SCOP (Figure 3A). However, no difference was observed in the STL of the acquisition trial (Day 1 of the passive avoidance test) among the control, SCOP-treated, and *C. chinensis* or *C. campestris* extract-treated groups (Figure 3A). SCOP increased the ratio of the transfer latency (TL) of the retention session to the TL of the acquisition session in the elevated plus-maze (Figure 3B). The *C. chinensis* or *C. campestris* extract at 100 and 300 mg/kg decreased the ratio of the TL of the retention session to the TL of the acquisition session that was increased by SCOP (Figure 3B).

**Figure 4:**
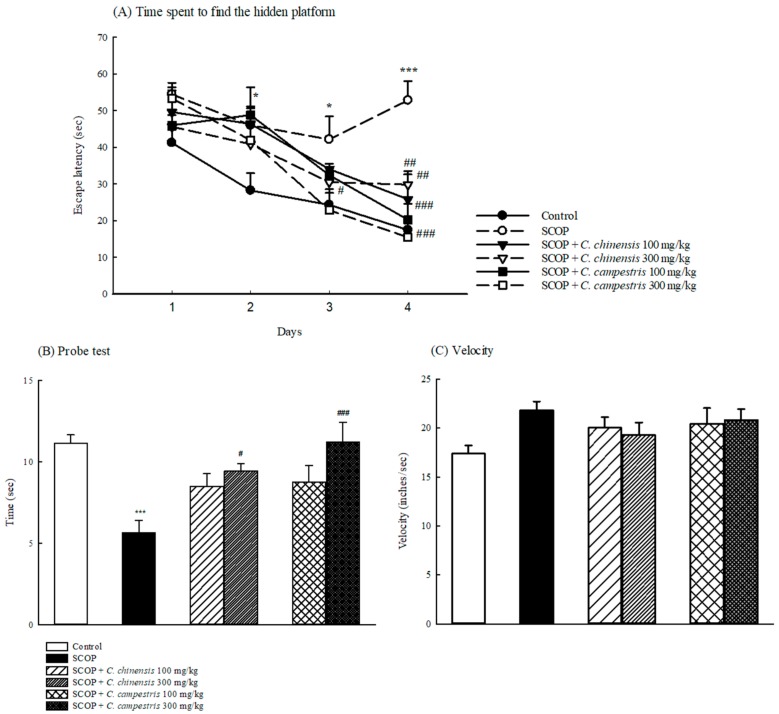
The effects of *Cuscuta chinensis* and *C. campestris* extracts (100 and 300 mg/kg, po) on (**A**) swimming time on Days 1–4 of MWM, (**B**) probe test of MWM, and (C) swimming velocity in scopolamine (SCOP, 1 mg/kg, ip)-injected mice. Columns indicate mean ± SEM (*n* = 7). * *p* < 0.05, *** *p* < 0.001 compared with the control group. # *p* < 0.05, ## *p* < 0.01, ### *p* < 0.001 compared with the SCOP group.

Mice injected with SCOP took a longer escape latency to reach the platform in the spatial performance test (Days 2–4 of the Morris water maze (MWM)) than those in the control group (Figure 4A). The *C. chinensis* extract at 100 and 300 mg/kg only shortened the longer escape latency, which was caused by SCOP, taken to reach the platform on Day 4 of the spatial performance test in MWM. The *C. campestris* extract at 100 and 300 mg/kg shortened the longer escape latency, which was caused by SCOP, taken to reach the platform on Days 3 and 4 of the spatial performance test in MWM (Figure 4A). Moreover, compared with the control group, SCOP decreased the time spent on the platform in the probe test of MWM (Figure 4B). The *C. chinensis* or *C. campestris* extract only at 300 mg/kg increased the time spent on the platform in the probe test of MWM (Figure 4B). Furthermore, SCOP accelerated the swimming velocity compared with that in the control group (Figure 4C). Compared with the SCOP group, SCOP-injected groups treated with the *C. chinensis* or *C. campestris* extract did not alter the swimming velocity in MWM (Figure 4C).

### 2.3. Effects of C. chinensis and C. campestris Extracts on Brain Acetylcholinesterase Activities in SCOP-Treated Mice

**Figure 5:**
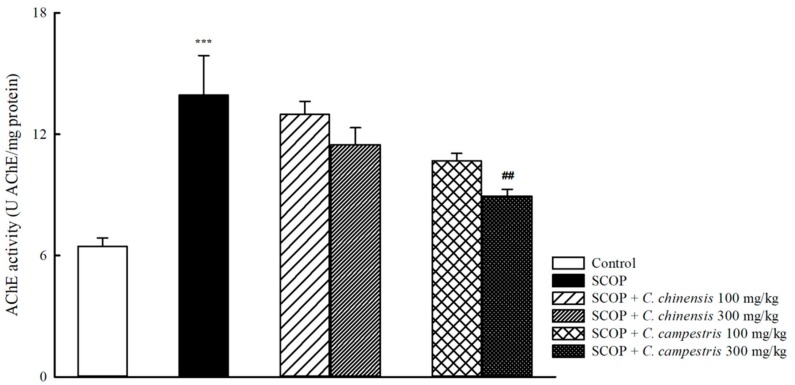
The effects of *Cuscuta chinensis* and *C. campestris* extracts (100 and 300 mg/kg, po) on brain acetylcholinesterase (AChE) activities in scopolamine (SCOP, 1 mg/kg, ip)-treated mice. All the brain tissues were collected from the tested mice and then homogenized to obtain the total brain homogenates. AChE activities in the total brain homogenates were subsequently examined. Columns indicate mean ± SEM (*n* = 7). *** *p* < 0.001 compared with the SCOP group. ## *p* < 0.01 compared with the SCOP group.

SCOP increased brain acetylcholinesterase (AChE) activities in mice (Figure 5). The *C. chinensis* extract at any dose did not alter brain AChE activities that were increased by SCOP. The *C. campestris* extract at 300 mg/kg decreased brain AChE activities that were increased by SCOP (Figure 5).

### 2.4. Effects of C. chinensis and C. campestris Extracts on Brain Oxidative Damage in SCOP-Treated Mice

**Figure 6:**
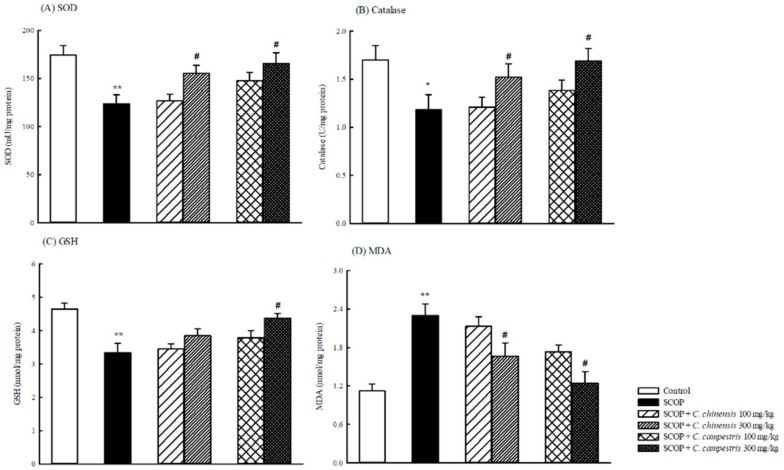
The effects of *Cuscuta chinensis* and *C. campestris* extracts (100 and 300 mg/kg, po) on (**A**) brain superoxide dismutase (SOD) activities, (**B**) brain catalase activities, (**C**) brain glutathione (GSH) levels, and (**D**) brain malondialdehyde (MDA) levels in scopolamine (SCOP, 1 mg/kg, ip)-treated mice. All the brain tissues were collected from the tested mice and then homogenized to obtain the total brain homogenates. The SOD and catalase activities and the GSH and MDA levels in the total brain homogenates were subsequently examined. Columns indicate mean ± SEM (*n* = 7). * *p* < 0.05, ** *p* < 0.01 compared with the control group. # *p* < 0.05 compared with the SCOP group.

SCOP decreased SOD and catalase activities and glutathione (GSH) levels and then increased MDA levels in the brains of mice (Figure 6). The *C. chinensis* or *C. campestris* extract at 300 mg/kg increased brain SOD and catalase activities that were decreased by SCOP. The *C. chinensis* or *C. campestris* extract at 300 mg/kg decreased MDA levels that were elevated by SCOP (Figure 6). However, only the *C. campestris* extract at 300 mg/kg increased brain GSH levels that were decreased by SCOP.

### 2.5. Effects of C. chinensis and C. campestris Extracts on Brain Cytokine Levels in SCOP-Treated Mice

**Figure 7:**
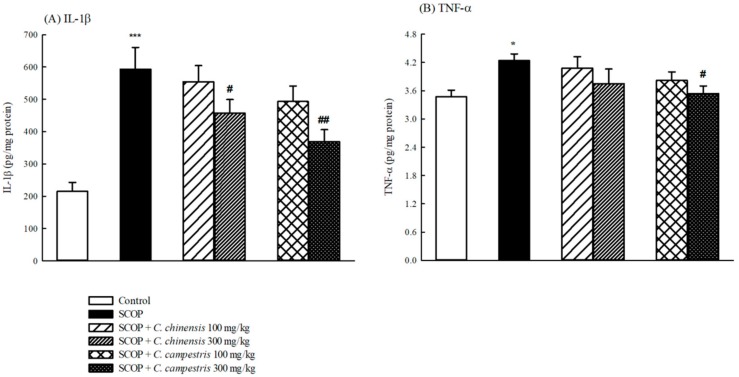
The effects of the *Cuscuta chinensis* and *C. campestris* extracts (100 and 300 mg/kg, po) on (**A**) brain interleukin-1β (IL-1β) levels and (**B**) brain tumor necrosis factor α (TNF-α) levels in scopolamine (SCOP, 1 mg/kg, ip)-treated mice. All the brain tissues were collected from the tested mice and then homogenized to obtain the total brain homogenates. The IL-1β and TNF-α levels in the total brain homogenates were subsequently examined. Columns indicate mean ± SEM (*n* = 7). * *p* < 0.05, *** *p* < 0.001 compared with the control group. # *p* < 0.05, ## *p* < 0.01 compared with the SCOP group.

SCOP increased brain interleukin-1β (IL-1β) and tumor necrosis factor-α (TNF-α) levels in mice (Figure 7). The *C. chinensis* extract at 300 mg/kg decreased brain IL-1β levels that were increased by SCOP injection (Figure 7). The *C. campestris* extract at 300 mg/kg decreased brain IL-1β and TNF-α levels that were increased by SCOP injection (Figure 7).

### 2.6. AChE-Inhibiting Activities of C. chinensis and C. campestris In Vitro

**Figure 8:**
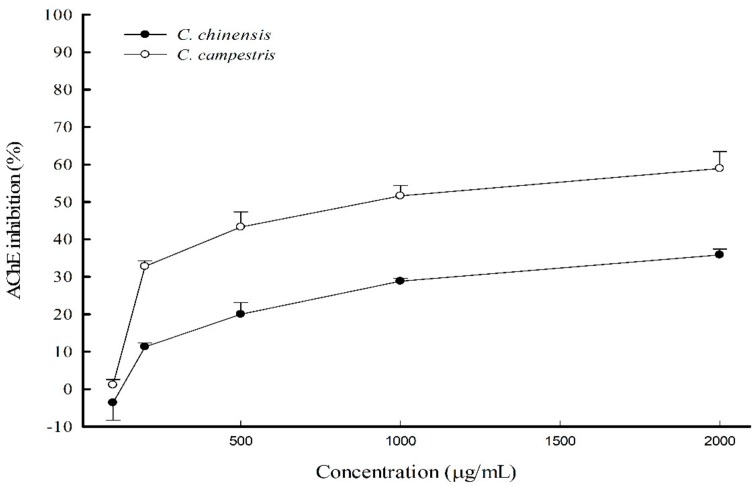
The acetylcholinesterase (AChE)-inhibiting effects of *Cuscuta chinensis* and *C. campestris* extracts in vitro. The supernatants of the brain homogenates were used as the AChE source for the assay. Four concentrations of the *C. chinensis* or *C. campestris* extract (0.5, 1, 1.5, and 2 mg/mL) were used to examine their inhibitory activities. Data are expressed as mean ± SD (*n* = 3).

The AChE-inhibiting activities of *C. chinensis* and *C. campestris* were examined using the Ellman method. The results showed that *C. chinensis* and *C. campestris* extracts at 100–2000 μg/mL possess AChE-inhibiting activities, and *C. campestris* has a higher inhibitory activity (Figure 8).

### 2.7. Antioxidant Compounds and Activity of C. chinensis and C. campestris In Vitro

The total flavonoid content of *C. chinensis* and *C. campestris* extracts was determined to be 20.07 ± 0.22 and 28.34 ± 0.95 mg/g, respectively, which is equivalent to that of quercetin. Thus, *C. campestris* has higher flavonoid content than *C. chinensis*. The content of total phenolic compounds of *C. chinensis* and *C. campestris* extracts was 76.78 ± 1.58 and 65.11 ± 0.21 mg/g, respectively, which is equivalent to that of gallic acid. Thus, *C. chinensis* has a higher content of phenolic compounds than *C. campestris* (Table 1). Furthermore, the antioxidant capacities of *C. chinensis* and *C. campestris* extracts were examined through the 2,2′-azino-bis(3-ethylbenzothiazoline-6-sulphonic acid) (ABTS) decolorization assay and the diphenyl picrylhydrazyl (DPPH) scavenging assay. The results showed that they possess an antioxidant capacity, and *C. chinensis* exhibits a slightly higher activity than *C. campestris* (Table 1).

## 3. Discussion

Traditional Chinese physicians commonly use *C. chinensis* as a herb for preventing aging. Early reports have indicated that *C. chinensis* improved memory impairment in the Y-maze or step-down avoidance test in aged, cerebral ischemia, or d-galactose-induced mice [22,23,24]. Due to the important role of the cerebral cholinergic system in memory function and AD, we newly discovered the memory-improving effects of *C. chinensis* and *C. campestris* on SCOP-induced memory deficit in mice and compared their efficacies simultaneously. In this study, this behavioral alteration caused by SCOP in mice was consistent with that described the previous reports in rodents [10,25,26], indicating that SCOP-induced memory deficit in the passive avoidance test, elevated plus-maze, and MWM is related to hyperactivity (hyperlocomotion), poor attention (poor exploratory behavior), and subsequent learning acquisition impairment (poor memory function). The *C. chinensis* extract at 300 mg/kg improved the passive avoidance response, memory index in the elevated plus-maze, and spatial performance and reference memory in MWM in mice, which were impaired by SCOP. These results of the memory-improving effects of the extracts are consistent with the finding of other reports that *C. chinensis* restores memory function in various models such as models of d-galactose aging, natural aging, cerebral ischemia, and hypoendocrinism induced by removing bilateral ovaries [17,22,23,24]. The *C. campestris* extract at 300 mg/kg also improved these aforementioned memory deficits that were impaired by SCOP in mice. The memory-improving effect of the *C. campestris* extract on SCOP-induced memory deficits is slightly better than that of the *C. chinensis* extract in mice. However, neither the *C. chinensis* nor *C. campestris* extracts altered hyperactivities and poor attention that were caused by SCOP. Therefore, *C. campestris* exerts memory-improving effects on SCOP-induced memory deficits, and these two species mainly improve the memory deficit but not the behavioral alteration caused by SCOP.

According to the neuropathological characterization of AD, cerebral oxidative stress and neuroinflammation are two major pathological factors that cause neuronal loss, particularly the degeneration of cholinergic neuronal circuits in BF [1]. Although SCOP is a muscarinic receptor antagonist, recent reports have indicated that SCOP causes oxidative damage and neuroinflammation in the cortex and hippocampus [10,11]. SCOP elevates oxidative modifications of proteins and lipids such as MDA and decreases the total antioxidant capacity in the brain [27]. SCOP also leads to the production of inflammatory factors, including IL-1β and TNF-α, in the brain [28]. The present study demonstrated that SCOP decreased the activities of antioxidant enzymes, including SOD and catalase, and GSH levels in the brains of mice and then increased brain MDA levels. In addition, SCOP increased IL-1β and TNF-α levels in the brains of mice. The present data further indicate that SCOP elevates AChE activities in the brains of mice. Consistent with previous reports [10,11,27], SCOP-induced memory deficits are strongly related to cholinergic dysfunction such as the upregulation of AChE activity and the blockade of muscarinic receptors, cerebral oxidative stress, and neuroinflammation. Some reports have stated that *C. chinensis* or *C. campestris* protects against oxidative damage caused by H_2_O_2_, acetaminophen, or CCl_4_ in vitro and in vivo [20,29,30]. *C. chinensis* or *C. campestris* possesses anti-inflammatory effects against LPS-induced or carrageenan-induced inflammation in vitro and in vivo [13,15,16]. The present study further explored the memory-improving mechanism of *C. chinensis* and *C. campestris* for SCOP-induced memory-impairment in mice. *C. chinensis* and *C. campestris* extracts only at 300 mg/kg restored the lower activities of the brain antioxidant defense system and decreased the higher brain MDA levels caused by SCOP in mice. *C. chinensis* and *C. campestris* extracts only at 300 mg/kg also decreased the higher brain IL-1β levels caused by SCOP in mice. Only the *C. campestris* extract at 300 mg/kg could restore brain GSH levels and decrease brain TNF-α levels in SCOP-injected mice. Only the *C. campestris* extract at 300 mg/kg inhibited the brain AChE activities upregulated by SCOP in mice. Furthermore, the present study also demonstrated that *C. campestris* possessed a higher AChE-inhibiting activity than *C. chinensis* in vitro. From these aforementioned results, we confirm that *C. chinensis* and *C. campestris* can improve the memory impairment caused by SCOP by restoring cholinergic function and decreasing oxidative stress and neuroinflammation. *C. campestris* has a higher memory-improving potential than *C. chinensis* against SCOP-induced memory impairment.

Previous studies have shown that seeds of both *C. chinensis* and *C. campestris* possess anti-inflammatory and antiproliferative activities, and the seeds of *C. campestris* exhibit a higher activity [16]. In this study, we demonstrated that *C. campestris* has a higher flavonoid content than *C. chinensis*, whereas *C. chinensis* has a higher content of phenolic compounds than *C. campestris*. The seeds of *C. chinensis* exhibit a higher antioxidant capacity than those of *C. campestris*. Therefore, the phenolic compounds in *Cuscuta* seeds make great contributions to the antioxidant capacity. However, *C. campestris* exhibits higher anti-inflammatory, antiproliferative [16], and neuroprotective bioactivities. Moreover, *C. campestris* has a higher content of flavonoids than *C. chinensis*. Therefore, these findings indicate that the flavonoids in the phenolic compounds make great contributions for the above bioactivities. Interestingly, quercetin accumulates at a dramatically higher level (about 2.14 mg/g) in the seeds of *C. campestris* than in *C. chinensis* [16]. In addition, quercetin possesses higher AChE-inhibiting potency in vitro and augments manganese-induced neurobehavioral alteration through the restoration of brain antioxidant status and AChE activity in rats [31,32,33]. These results indicate that quercetin may play a critical role in neuroprotective bioactivity in vitro and in vivo.

## 4. Materials and Methods

### 4.1. Preparation of Plant Extract

**Figure 9:**
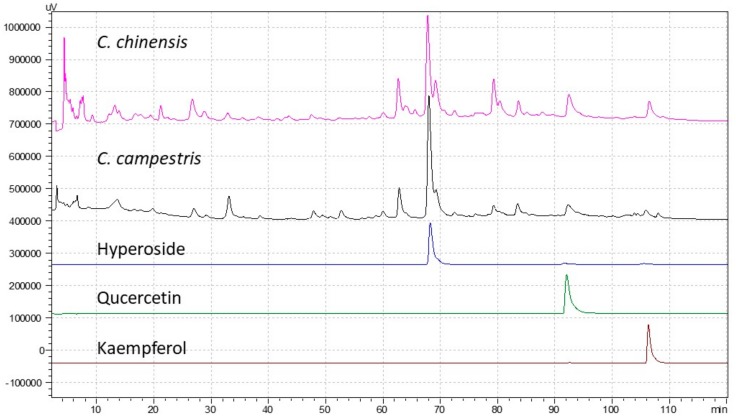
The HPLC profiles of *C. chinensis* and *C. campestris*. Phytochemical analyses of *C. chinensis* and *C. campestris* methanol extracts, as well as three standards, were performed using a Shimadzu HPLC system with an RP-18 column at UV 254 nm.

*C. chinensis* seeds were purchased from a Chinese medicine hospital (Union Traditional Medical Hospital, Taichung, Taiwan). *C. campestris* seeds were collected from *C. campestris* plants grown on *Bidens pilosa* L. var. *radiata* Sch. Bip. in the wild (Taichung, Taiwan). The seeds of both species were authenticated by Wen-Huang Peng and Ming-Kuem Lin (both from the Department of Chinese Pharmaceutical Sciences and Chinese Medicine Resources, China Medical University, Taichung, Taiwan), who observed the morphology of the flowers and confirmed the chemical compositions of the seeds [13,16,20]. The raw seeds were processed as described previously [16]. To prepare methanol extracts, *Cuscuta* seeds were extracted with methanol through sonication, as described previously [16]. Phytochemical profiles of *C. chinensis* and *C. campestris* methanol extracts were determined and compared to confirm their identities (Figure 9).

### 4.2. Chemicals

The reagents 5,5′-dithio-bis(2-nitrobenzoic) acid (DTNB), ABTS, AChE, acetylthiocholine iodide (ACtCh), aluminum chloride, l-ascorbic acid, DPPH, Folin–Ciocalteu’s reagent, gallic acid, GSH, glutathione reductase, nitroblue tetrazolium chloride (NBT), hyperoside, quercetin, kaempferol, SCOP hydrobromide, SOD, sodium carbonate, trichloroacetic acid, thiobarbituric acid (TBA), tetraisopropyl pyrophosphoramide (iso-OMPA), xanthine oxidase, and xanthine were purchased from Sigma-Aldrich Chem. Corp. (St. Louis, MO, USA). 

### 4.3. HPLC Analysis

Samples and standards were resolved in methanol and filtered through a 0.22-mm membrane. HPLC was performed on a Shimadzu HPLC system (Kyoto, Japan) equipped with a Shimadzu LC-20AT pump, a Shimadzu SIL-20 autosampler, and a Shimadzu SPD-M20A detector (wavelength: 190–800 nm). The HPLC profiles of *C. chinensis* and *C. campestris* were determined using an RP-18 column (Cosmosil C18, 4.6 × 250 mm, 5 mm, Nacalai Tesque Inc., Kyoto, Japan) at a flow rate of 1.0 mL/min, detected at UV 254 nm. The injection volume was 10 μL. The mobile phase was composed of 0.1% TFA water solution (solvent A) and methanol (solvent B). The solvent gradient was as follows: 0–120 min from 10% to 60% solvent B.

### 4.4. Animals

Eight-week-old male ICR mice (20–25 g) were obtained from BioLASCO Taiwan Co., Ltd. (Yi-Lan, Taiwan). They were housed randomly in groups of six at a temperature of 23 °C ± 1 °C, 60% humidity, and 12/12-h light/dark cycle (light phase: 08:00 to 20:00). These mice were maintained following the Guiding Principles for the Care and Use of Laboratory Animals, and the experimental protocol was approved by the Institutional Animal Care and Use Committee of China Medical University (CMUIACUC-2017392). After acclimatization for 1 week, normal mice were used in the experiments mentioned below.

### 4.5. Drug Treatment and Group Division

**Figure 10:**
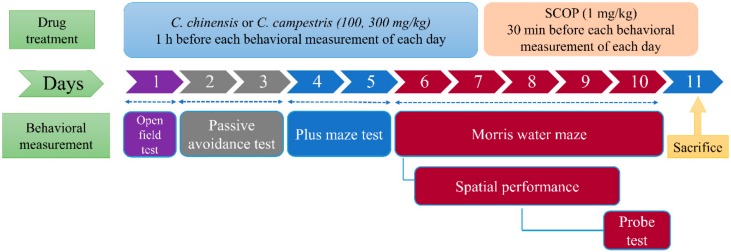
The schedule of drug treatments and behavioral measurements. The *Cuscuta chinensis* or *C. campestris* extract (100 and 300 mg/kg, po) was administered 1 h before each behavioral assessment of each day during the experimental period. Scopolamine (SCOP, 1 mg/kg, ip) was administered 1 h before each behavioral assessment of each day during the experimental period. *C. chinensis*: *Cuscuta chinensis*; *C. campestris*: *Cuscuta campestris*.

The schedule of drug treatments and behavioral measurements is shown in Figure 10. SCOP (1 mg/kg) was intraperitoneally (ip) injected into mice 30 min before behavioral measurements [26]. The *C. chinensis* or *C. campestris* extract (100 and 300 mg/kg) was orally administered to mice 1 h before behavioral measurements. Behavioral measurements were performed in the following order: locomotor and exploratory tests, passive avoidance test, elevated plus-maze, and spatial performance test and probe test of MWM. On the next day, the probe test was conducted, and the tested mice were sacrificed 1 h after the test. The activities of AChE and antioxidant enzymes (SOD and catalase) and the levels of GSH, MDA, and cytokines (IL-1β and TNF-α) were monitored in the brain tissues of the tested mice.

### 4.6. Locomotor and Exploratory Tests

The open field apparatus (Coulbourn Instruments L.L.C., Holliston, MA, USA) was used to perform the locomotor and exploratory tests simultaneously. Each mouse was placed in the open field apparatus; then, the movement time, distance, velocity, number of entries, and time spent in the hole were recorded for 10 min by using the TruScan software v 2.07 (Coulbourn Instruments L.L.C., Holliston, MA, USA) [25].

### 4.7. Passive Avoidance Response

Two compartments (light and dark compartments) of the same size that had a steel-rod grid floor (Coulbourn Instruments L.L.C., Holliston, MA, USA) were used to perform the passive avoidance test. A guillotine door was located between the two compartments. In the beginning, each mouse was placed in the light compartment; then, the STL was measured by recording the time when the door was opened and the mouse entered the dark compartment. The door was locked once the mouse entered the dark compartment. Subsequently, the mouse received a footshock through the steel rods. The mouse was placed back its home cage 5 s after the shock. After 24 h, the same experiment was conducted again, and the STL for the second day was recorded [26].

### 4.8. Elevated Plus-Maze

Two enclosed arms and two open arms were connected with each other to form a plus-maze. The plus-maze was elevated to a height of 0.5 m above the floor. In the acquisition session, each mouse was placed at the distal end of an open arm, and the TL was recorded. After entering the enclosed arm, the mouse was free to move in the maze for 10 s. Subsequently, the mouse returned to its home cage. The retention session was performed 24 h after the acquisition session [34]. The TL in the acquisition and retention sessions were recorded by a video camera equipped with an automated video tracking system device and EthoVision XT software (version 10, Noldus Information Technology, Leesburg, VA, USA).

### 4.9. MWM

A black circular stainless pool containing water (23 °C ± 1 °C) to a depth of 35 cm and a hidden platform submerged 1.0 cm below the water was used for MWM. In four sessions for 4 days, each mouse was trained each day to find the hidden platform in the spatial performance test. During the training, the mouse facing the pool wall was placed in the water and was allowed to find the platform within 60 s. If the mouse reached the platform successfully, it was allowed to rest for 15 s on the platform. If it could not reach the platform within 60 s, the mouse was given a score of 60 s, was moved manually to the platform and was allowed to rest for 15 s on the platform. Each mouse was trained four times. The swimming path and escape latency to the platform were also recorded using the aforementioned video camera [25]. On the next day, the platform was removed, and the reference memory was then measured by performing the probe test. In addition, each mouse was placed into the pool at the location opposite to the previous location. The distance moved and time spent by each mouse in each quadrant was recorded during the 60 s [25].

### 4.10. Brain Tissue Preparation

All mice were anesthetized with sodium pentobarbital (50 mg/kg) and were sacrificed through decapitation in an unconscious state after behavioral measurements. All brain tissues were collected and homogenized with 9× (*w*/*v*) ice-cold phosphate buffered saline. The homogenates were centrifuged at 10,000× *g* for 15 min at 4 °C, and the aliquots of supernatants were stored at −80 °C until the examination of AChE and antioxidant enzyme activities and GSH and MDA levels.

### 4.11. Measurement of Brain AChE Activities

Brain AChE activity was determined through a method described in a previous report [25]. The supernatant solution or AChE standard solution was incubated with DTNB at 25 °C for 10 min; then, 0.3 mM ACtCh was added for color development. The yellow product was measured at 412 nm using a spectrophotometric microplate reader (PowerWave X340, Bio-Tek instruments, Inc., Winooski, VT, USA). Brain AChE activity is expressed as U AChE/mg of protein.

### 4.12. Measurement of Brain Antioxidant Enzyme Activities

Catalase and SOD activities and GSH and MDA levels were determined using a spectrophotometric microplate reader [35]. Catalase activity was examined by measuring the absorbance of Amplex Red at 560 nm. SOD activity was determined by measuring the absorbance of NBT at 560 nm. Catalase and SOD activities were expressed as U/mg of protein. The GSH level was determined by measuring the absorbance of DTNB at 405 nm. The GSH levels are expressed as nmol/mg of protein. Brain MDA levels were determined through the TBARS assay. The absorbance of TBA was determined at 532 nm. MDA levels are expressed as nmol/mg of protein.

### 4.13. Measurement of Brain IL-1β and TNF-α Levels

The brain tissues were homogenized with a lysis solution containing protease inhibitors (0.4 M NaCl, 0.1 mM phenylmethylsulfonyl fluoride, 0.1 mM benzethonium chloride, 10 mM EDTA, 0.5% bovine serum albumin, 0.05% Tween 20, and 10 μg/mL aprotinin). Homogenates were centrifuged at 10,000× *g* at 4 °C, and the supernatants were stored at −80 °C until the following analyses. The levels of TNF-α and IL-1β proteins were determined using ELISA kits (R&D Systems, Abingdon, UK) according to the manufacturer’s protocol.

### 4.14. In Vitro AChE-Inhibiting Activity Assay

The assay of AChE-inhibiting activity was conducted as described in a previous report [26]. The mouse brain was dissected on ice and homogenized. The brain homogenate was centrifuged, and the supernatant was used as the AChE source for the assay. Four concentrations of the *C. chinensis* or *C. campestris* extract (0.5, 1, 1.5, and 2 mg/mL) were used to examine their inhibitory activities. The reaction mixture was placed in a Bio-Teck PowerWave 340X microplate reader (Bio-Tek instruments, Inc., Winooski, VT, USA) to record the absorbance at 412 nm. The percentage of AChE inhibition was calculated by comparing the rates of the samples to those of the solvent.

### 4.15. Measurement of Antioxidant Phytoconstituent Contents and Activity In Vitro

The total flavonoid content in the extract was determined using the aluminum chloride colorimetric assay [35]. The content of the total phenolic compounds in the extract was estimated using the Folin-Ciocalteu method [35]. The ABTS decolorization assay and DPPH scavenging assay were used to measure the antioxidant capacity in comparison with the standard l-ascorbic acid [35].

### 4.16. Statistical Analyses

All values are expressed as the mean ± standard error of the mean (SEM). IBM SPSS version 20.0 (IBM, Chicago, IL, USA) was used to analyze all the results through one-way ANOVA followed by Scheffe’s test. A *p* value of < 0.05 was considered significant.

## 5. Conclusions

In Chinese medicine pharmacopoeias, Cuscutae Semen mainly refers to the seeds of *C. chinensis* Lam. According to the findings of previous reports for anti-inflammatory and antiproliferative activities [16] and the present results for antioxidant and neuroprotective capacities, the seeds of *C. campestris* exhibit the same bioactivities as those of *C. chinensis*. The memory-improving mechanism against SCOP-induced memory impairment is mainly entered by restoring the cholinergic function and decreasing oxidative stress and neuroinflammation. Therefore, the seeds of *C. campestris* can be used as Cuscutae Semen in Traditional Chinese Medicine.

## Figures and Tables

**Table 1:** The contents of total flavonoids and total phenolic compounds in the *Cuscuta chinensis or C. campestris* extract and their ABTS antioxidant capacity and DPPH scavenging ability.

	Contents of Antioxidant Compounds		Radical Scavenging Ability l-Ascorbic Acid Equivalent (μg/mL)	
	Flavonoids (mg quercetin equivalent/g)	Total phenolics (mg gallic acid equivalent/g)	ABTS	DPPH
*C. chinensis*	20.07 ± 0.22	76.78 ± 1.58	27.87 ± 3.46	11.18 ± 0.46
*C. campestris*	28.34 ± 0.95	65.11 ± 0.21	26.48 ± 3.26	10.80 ± 0.48

Data are expressed as mean ± SD (*n* = 3). ABTS: 2,2′-azino-bis(3-ethylbenzothiazoline-6-sulphonic acid), DPPH: diphenyl picrylhydrazyl.

## Abbreviations

ABTS	2,2′-azino-bis(3-ethylbenzothiazoline-6-sulphonic acid)
AChE	acetylcholinesterase
ACtCh	acetylthiocholine iodide
AD	Alzheimer’s disease
BF	basal forebrain
DMSO	dimethyl sulfoxide
DPPH	diphenyl picrylhydrazyl
DTNB	5,5′-dithio-bis(2-nitrobenzoic) acid
GPx	glutathione peroxidase
GR	glutathione reductase
GSH	glutathione
iso-OMPA	tetraisopropyl pyrophosphoramide
MDA	malondialdehyde
MWM	Morris water maze
NBT	nitroblue tetrazolium chloride
SCOP	scopolamine
SOD	superoxide dismutase
TBA	thiobarbituric acid
TCA	trichloroacetic acid
